# Photosynthetic circadian rhythmicity patterns of *Symbiodium*, the coral endosymbiotic algae

**DOI:** 10.1098/rspb.2012.2942

**Published:** 2013-05-22

**Authors:** Michal Sorek, Yosef Z. Yacobi, Modi Roopin, Ilana Berman-Frank, Oren Levy

**Affiliations:** 1 The Mina and Everard Goodman Faculty of Life Sciences, Bar-Ilan University, Ramat-Gan 52900, Israel; 2 Kinneret Limnological Laboratory, Oceanographic and Limnological Research Ltd., Migdal, Israel

**Keywords:** circadian rhythm, photosynthesis, *Symbiodinium*, coral, oxygen evolution

## Abstract

Biological clocks are self-sustained endogenous timers that enable organisms (from cyanobacteria to humans) to anticipate daily environmental rhythms, and adjust their physiology and behaviour accordingly. Symbiotic corals play a central role in the creation of biologically rich ecosystems based on mutualistic symbioses between the invertebrate coral and dinoflagellate protists from the genus *Symbiodinium*. In this study, we experimentally establish that *Symbiodinium* photosynthesis, both as a free-living unicellular algae and as part of the symbiotic association with the coral *Stylophora pistillata*, is ‘wired’ to the circadian clock mechanism with a ‘free-run’ cycle close to 24 h. Associated photosynthetic pigments also showed rhythmicity under light/dark conditions and under constant light conditions, while the expression of the oxygen-evolving enhancer 1 gene (within photosystem II) coincided with photosynthetically evolved oxygen in *Symbiodinium* cultures. Thus, circadian regulation of the *Symbiodinium* photosynthesis is, however, complicated as being linked to the coral/host that have probably profound physiochemical influence on the intracellular environment. The temporal patterns of photosynthesis demonstrated here highlight the physiological complexity and interdependence of the algae circadian clock associated in this symbiosis and the plasticity of algae regulatory mechanisms downstream of the circadian clock.

## 1. Introduction

Life has evolved under rhythmic day and night cycles, caused by our planet's rotation, which control light and temperature. In response to these cycles, most organisms from cyanobacteria to mammals have developed endogenous clocks that enable them to anticipate daily and seasonal rhythms, and to adjust their biochemical, physiological and behavioural processes accordingly [1,2]. Environmental cues, particularly light and temperature, maintain the rhythm of the endogenous clock system. Subsequently, numerous circadian physiological responses have been identified in plants, including phototropic responses, hypocotyl elongation, cotyledon expansion, stomata opening, chloroplast development and the expression of specific genes [3–5]. Circadian oscillations have been observed in plants and in many algae for varying biological processes, including cell division, phototoxicity, motility, bioluminescence and sensitivity to ultraviolet light [6–10].

Photosynthesis is one of the most fundamental processes for maintaining life, with circadian control regulating different components of the photosynthetic apparatus in higher plants [11,12], in macroalgae and in groups of unicellular algae [13–16]. The marine unicellular algae *Lingulodinium*, the most intensively investigated dinoflagellate in the field of circadian rhythms, can maintain a diel periodicity of photosynthesis for several days and even weeks under conditions of constant dim light [17]. Circadian rhythms have also been identified in several other marine dinoflagellate species, including *Pyrocystis lunula* [18–20] and recently in the green unicellular coccoid, *Ostreococcus* [21,22].

Certain marine dinoflagellates form symbiotic associations with invertebrate hosts in both tropical and temperate oceans [23,24]. The most famous is *Symbiodinium* spp. (zooxanthellae), a diverse genus of symbiotic dinoflagellates with eight subgeneric clades, A–H [25,26], which are obligate symbionts of many invertebrates including coral species. The success of the association between *Symbiodinium* and coral is typified by the tightly coupled mutualistic interactions that allow *Symbiodinium* to supply valuable energy to the host [27–29]. However, much less is known about how the circadian system is integrated in marine symbiotic organisms, such as coral reefs, and how the photosynthetic apparatus is governed within this association. Studying the complexity of the circadian clock in these cnidarian–dinoflagellate symbioses can help us understand host–symbiont interactions at the cellular and the molecular levels.

This study demonstrates that photosynthesis of *Symbiodinium*, both as a free-living unicellular organism and as part of a symbiotic association with the coral *Stylophora pistillata*, possesses diel rhythmicity with a periodicity close to 24 h under light/dark (LD) cycles (12 L : 12 D) during the zeitgeber time (ZT), and a periodicity of 23.5–24.4 h under constant light (LL) during the circadian time (CT). We examined photosynthetic oxygen production and the yield of photosystem II (PSII) in relation to changes in light periodicity. In addition, we measured the rhythmic patterns of photosynthetic pigment concentrations and the expression of the oxygen-evolving enhancer 1 (*OEE1*) gene under 48 h of LL in cultured *Symbiodinium*. Our results provide new evidence that the circadian clock machinery governs the photosynthetic apparatus in unicellular symbiotic algae, with light as an important cue responsible for the duration of the free-run cycle. The results reveal a new complexity in the circadian rhythms of basal symbiotic organisms, such as reef-building corals, and demonstrate the ability of the algae to simultaneously tune their circadian machinery to respond to external cues and balance such as light.

## 2. Material and methods

### (a) Collection and maintenance of corals and *Symbiodinium* cultures

SCUBA divers collected the *S. pistillata* corals tested in this study from a depth of 10 m in front of the Interuniversity Institute for Marine Sciences, Eilat, Gulf of Aqaba, Red Sea, Israel. After collection, the corals were first placed in running sea water and then placed in 900 l aquaria for a period of one week for acclimation prior to the LD cycle experiments of 14 : 10 h. The artificial light cycles were achieved by illuminating the colonies with 200 µmol quanta m^−2^ s^−1^ using 400 W Aqualine 10 000 K metal halide lamps.

The *Symbiodinium* cultures used in this study were clade A1 (CCMP 2467), clade C1 (CCMP 2466) and clade D (CCMP 2556) in stationary stage growth. All cultures were grown in 2 l Fernbach flasks containing 1 l half-strength medium (f/2) without silica. Illumination was provided by lateral fluorescent lamps under a 12 L : 12 D cycle. The cultures were maintained in a culture room with a controlled temperature set to 24°C for all indoor experiments. Irradiance was measured by a quantum flat sensor (LI-COR, Lincoln, NE, USA) and found to be 100 µmol quanta m^−2^ s^−1^.

### (b) Oxygen evolution monitoring

*In situ* oxygen flux data were obtained from three coral colonies using a three-chamber submersible respirometre deployed in a 1 m, 900 l tank. This instrument is equipped with three 2.5 l chambers, an oxygen sensor (Kent EIL galvanic type ABB, Inc.), light metre, temperature probe and data logger [30]. The chamber contents were constantly stirred, and the water was changed every 20 min to prevent super-saturation of the sea water with oxygen. A total of 400 W Aqualine 10 000 K metal halide lamps provided artificial lighting. In the first 48 h of the experiment, colonies were subjected to LD cycles of 14 : 10 h; (ZT), then the colonies were illuminated under LL for 72 h (CT). During the experiments, the tank was shaded using a double black clove cover to avoid transmission of ambient light. The data were processed using the Australian Institute for Marine Science ‘Respiro’ programme to calibrate and normalize the data.

Oxygen evolution in cultured *Symbiodinium* was monitored with a four-channel oxygen metre (OXY4) equipped with four optical oxygen sensors (OPTODs; PreSens, Germany) connected to four dipping probes (DP-PSts). The OPTODs continuously monitored the oxygen evolution at intervals of 5 min. The three different clades (A, C and D) were monitored over a period of 9 days: 2 days on the LD cycle, followed by 4 days of LL; after the LL period, the cultures were introduced again to the normal LD cycle for recovery measurements for another 3 days. For each experiment, four cultures were tested; prior to the experiments, the oxygen in medium-only (free of algae) flasks were measured to remove any potential artefacts from the OXY-4 probe drifting, f/2 medium and temperature.

### (c) Photosystem II measurements

The maximum quantum yield for cultured *Symbiodinium* clade A (in the same growing conditions as in the pigment analysis) was sampled at 4 h intervals over 2 days of 12 L : 12 D cycles, followed by 2 days of LL. A 5 ml culture was sampled at each time point and placed in the dark for 30 min. The sample was then analysed using a fluorescence induction and relaxation system (FIRe, Satlantic, Halifax, Canada; [31]). The biophysical principles were based on the fast repetition rate fluorometry and detailed in Kolber *et al.* [32]. The FIRe was set to deliver saturation flash sequences of one hundred 1 ms flashes with 1 ms intervals between flashes. Acquisitions of a series of three flash sequences were internally averaged. The maximum quantum yield (*F*_v_/*F*_m_) was calculated after blanks (0.2 μm filtered f/2 medium in artificial sea water) were subtracted from the initial, dark-adapted fluorescence (*F*_0_) and the maximal fluorescence (*F*_m_) when all PSII reaction centres were photochemically reduced for each sample. Functional absorption cross section of PSII (*σ*PSII) was calculated as well. The coral maximum quantum yield was measured using the imaging-pulse-amplitude-modulation (PAM) method (Maxi-PAM, Walz Gmbh, Effeltrich, Germany). In this experiment, corals were kept under LL conditions and measured during CT from 06.00 (CT 0) until 20.00 (CT 14). The coral branches were analysed at CT 0, 2, 6, 10 and 14. Prior to analysis, the branches were maintained in the dark for 30 min and transferred to the imaging PAM machine for analysis. The resulting images were analysed using the Imaging-Win software (v. 2.00m; Walz Gmbh) and recorded for each of the branches. The dark-level fluorescence yield (*F*_0_) and the maximum fluorescence yield (*F*_m_) were determined, and the maximum quantum yield (*F*_v_/*F*_m_) was calculated. We performed light-response curves with increasing illuminations of 20 s intervals (0, 1, 16, 41, 81, 141, 221, 276, 351, 426, 501, 601, 701, 801 and 901 µmol quanta m^−2^ s^−1^) and calculated the electron transport rate (ETR).

### (d) Pigment analysis

*Symbiodinium* clade A cultures were grown in the laboratory under 100 µmol quanta m^−2^ s^−1^ at 24°C and sampled at 4 h intervals during 2 days of LD cycles followed by 2 days of LL. Pigment concentrations were determined using 50 ml culture samples filtered onto glass fibre filters (Whatman GF/F 25 mm ± 0.7 µm). The filters were kept at −20°C in the dark until analysis. Extractions were performed in cold 100 per cent acetone and kept overnight in the dark at 4°C followed by ultrasonic disruption. The reverse-phase high-performance liquid chromatography (HPLC) system and standards followed the protocol of Yacobi *et al.* [33]. Cell pigment contents (chlorophyll *a*, chlorophyll *c*, peridinin, diadinoxanthin and diatoxanthin) were calculated by normalizing the HPLC concentration to cell number counts.

### (e) Oxygen-evolving enhancer 1 gene expression and quantification

Cultured algae from clade A were grown at 24°C under 100 µmol quanta m^−2^ s^−1^ and were sampled every 4 h for 4 days during a 48 h LD cycle (12 L : 12 D) followed by 2 days of LL. For RNA extractions, 50 ml of *Symbiodinium* culture was collected by centrifugation (6500 r.p.m. for 5 min at 4°C), and cell pellets were re-suspended in 500 µl of acidic phenol (Sigma) with 500 µl of NAES extraction buffer (50 mM sodium acetate buffer, 10 mM EDTA and 1% SDS, pH 5.0) and 100 mg of glass beads (less than 106 µm average diameter; Sigma). The mixture was vortexed for 1 min and immediately incubated for 5 min at 65°C. The process was repeated twice, and the protocol of Tu *et al.* [34] was then followed. RNA quantity and integrity were assessed with a NanoDrop ND-1000 spectrophotometre and an Agilent 2100 Bioanalyzer, respectively. RNA with an integrity number of greater than 9 was kept at −80°C for the next stage of qPCR analysis. The extracted RNA was amplified using MessageAmp II mRNA amplification kit (Ambion) to achieve an average of 20 μg RNA per extract. cDNA was synthesized using 1 µg of total RNA extracted with Verso cDNA synthesis kit (Thermo Scientific), following the kit's protocol. Primers were designed with Primer Quest software (Integrated DNA Technologies) for the *OEE1* gene based on expressed sequence tag data from KB8 Symbiodinium—clade A established by the Monica Medina group at the University of California, Merced (data can be found at the following website: http://medinalab.org/zoox). The obtained transcriptomes were sequenced by the Joint Genome Institute, and the raw data were deposited in the Short Read Archive at NCBI:

R: 5′-TGTGCTGCTGAAGGGCATCTTCTA-3′
F: 5′-TTTATCGCAGCGTTGCTGTCCTCA-3′
For *PsbA* our reference gene, we used the following primers:
R: 5′-AGCAAATGCAGCAACTACTGGAGC-3′
F: 5′-CTTCACTTCATGCTTGGTGTGGCT-3′

qPCR reactions were performed using the Corbett RG6000 real-time detection system with GoTaq qPCR Master Mix. Each 20 μl reaction contained 5 μl of GoTaq, 0.25 μl each of 10 mM forward and reverse primer, 1.5 μl of distilled deionized water and 3 μl of diluted cDNA (1 : 200). The following thermal profile was used: 94°C for 7 min followed by 45 cycles of 94°C for 7 s, 60°C for 15 s and 72°C for 20 s. Runs were analysed using the Rotor-Gene 6000 series software 1.7. The fluorescent threshold was set at 0.02 for all runs. The *OEE1* gene and the *PsbA* gene were normalized against the housekeeping gene *cyclophin*, which showed the most stable expression pattern in our experiment, identical to the findings of Rosic *et al.* [35]. The relative expression of each sample and gene was calculated using the method of 2^−*ΔΔ*CT^.

### (f) Data analysis

Oxygen data sampled on the time domain was transformed into the frequency domain via Fourier transformation. For this purpose, we coded an application in Python environment using the ‘scipy’ library function ‘fft’. From the output of the application, we extracted the dominant frequencies that characterize the examined rhythm. For statistical analysis, paired *t*-tests and one-way ANOVA followed by Tukey honestly significant difference (HSD) were used to assess the differences in experimental treatments of oxygen periodicity and gene expression under different light measurements. All statistical analyses were conducted using SPSS v. 20.0 (IBM, USA), and the results were considered statistically significant at *p* < 0.05.

## 3. Results

**Figure 1.**
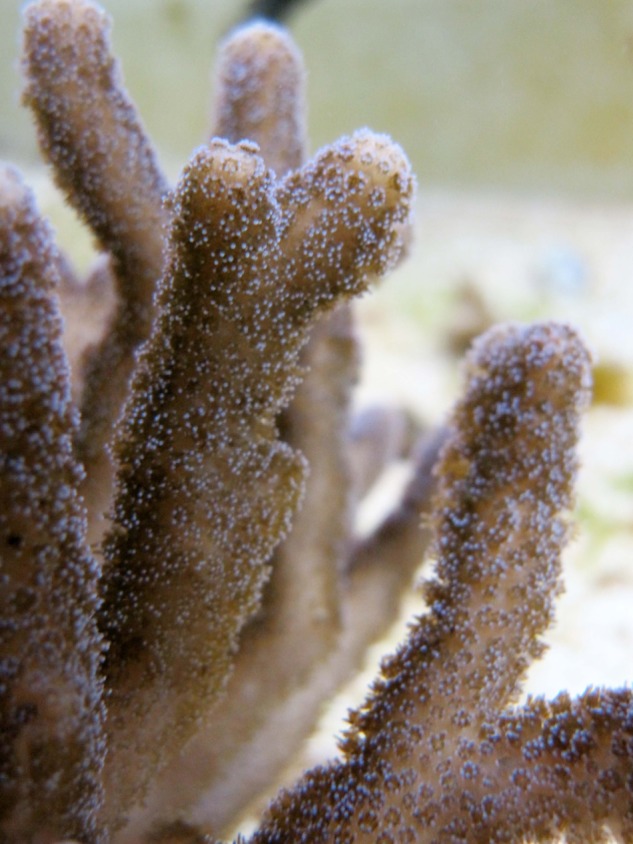
The symbiotic coral *S. pistillata* collected from the Red Sea at 5 m depth and used in this study.

**Figure 2.**
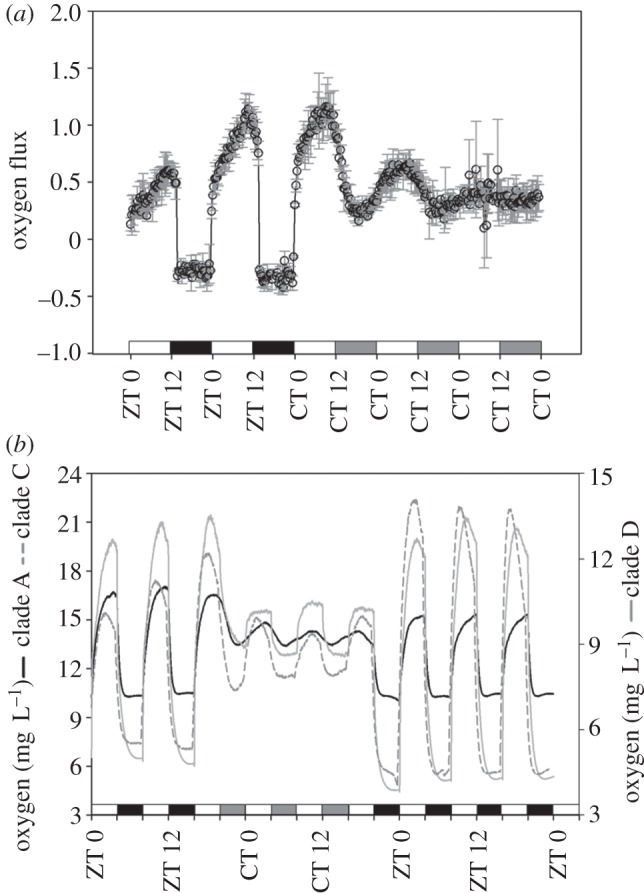
Oxygen evolution measurements. (*a*) Measurements of oxygen flux measured on *Symbiodinium* algae embedded in the Red Sea symbiotic coral *S. pistillata* (each time point, *n* = 3) during 2 days of LD cycles—zeitgeber time (ZT) followed by 3 days of LL—circadian time (CT) using a three-chamber submersible respirometre. (*b*) Oxygen evolution of *Symbiodinium* cultures three clades: A (black line), C (grey-dotted line) and D (grey line); for each time point, *n* = 4 during 3 days of LD cycles (12 L : 12 D) followed by 4 days of LL and 3 days of recovery with an LD periodicity of 12 L : 12 D. The cultures were monitored with a four-channel oxygen metre (OXY4). The white bars indicate light periods, the black bars indicate dark periods and the grey bars indicate subjective darkness.

Oxygen evolution was first recorded in the symbiotic reef-building coral *S. pistillata* (figure 1), which contains mostly type A and C *Symbiodinium* [36]. The measurements of oxygen evolution revealed that coral colonies maintained in running sea water for three consecutive days under LL (200 μmol m^−2^ s^−1^) showed rhythmic oscillations, with maximum photosynthetic rates occurring during the midday hours. Fourier transform analysis revealed a free-run cycle of 22.9 ± 0.4 h under LL. The amplitude dampened on the last day, an effect that did not occur under the normal LD oscillations (figure 2*a*).

**Figure 3.**
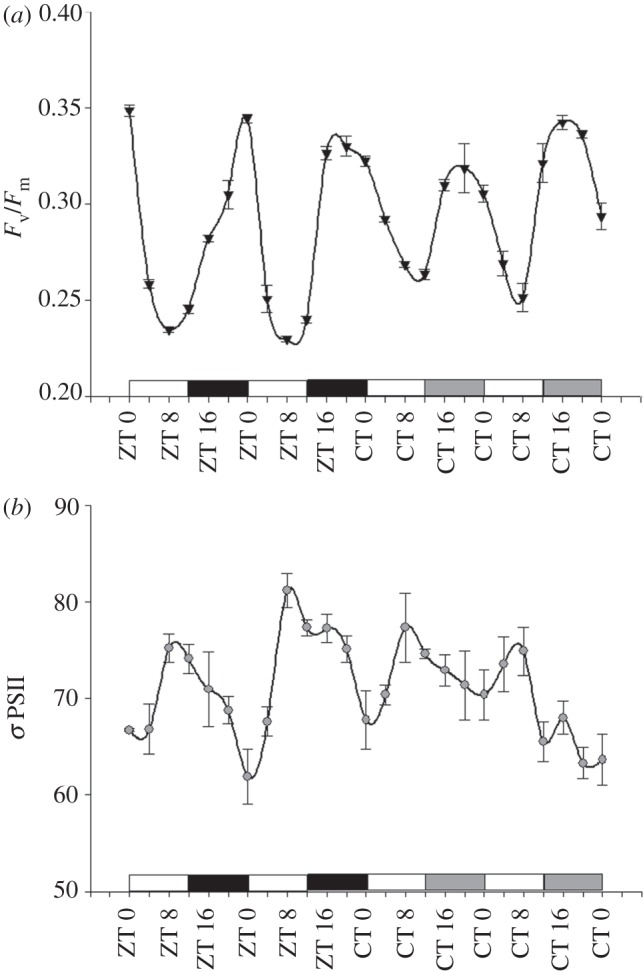
*Symbiodinium* chlorophyll fluorescence measurements. (*a*) F_v_/F_m_ values calculated using a FIRe fluorometre on *Symbiodinium* clade A cultures during 2 days of LD (12 L : 12 D) (ZT) followed by 2 days of LL—(CT). Inverted triangles denote *F*_v_/*F*_m_. (*b*) Sigma PSII values (grey circles) of clade A under the same conditions as (*a*). The white bars indicate light periods, the black bars indicate dark periods and the grey bars indicate subjective darkness (each time point, *n* = 3 ± s.d.).

**Figure 4.**
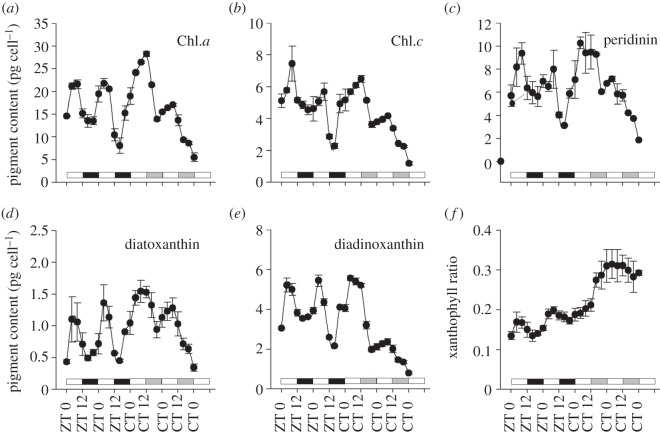
HPLC analysis of pigments. (*a*–*e*) Diel oscillations of pigments in *Symbiodinium* cultures (clade A), under LD (ZT) and LL (CT). (*f*) Calculated ratio of xanthophyll cycle carotenoids (diatoxanthin(/diatoxanthin + diadinoxanthin)). The white bars indicate light periods, the black bars indicate dark periods and the grey bars indicate subjective darkness (each time point, *n* = 3 ± s.d.).

To further investigate if photosynthesis has similar rhythmic oscillations in the free-living form of *Symbiodinium* without the influence of the coral host, three cultures, clades A, C and D, were tested. We used clade A and clade C because they mainly inhabit *S. pistillata* colonies in the Red Sea, and we decided to use clade D additionally in the experiments because it is considered to be more tolerant to high temperature and irradiance conditions [37]. Cultures which were grown under 12 L : 12 D cycles at 24°C demonstrated approximately the same clear pattern and time period of oxygen evolution in comparison with the coral measurements. Clades A and C had a cycle of 24.3 ± 0.23 h, which was significantly different from clade D (25.2 ± 0.3 h; one-way ANOVA followed by Tukey HSD, *p* < 0.05; figure 2*b*; electronic supplementary material, S1, S2). The maximum oxygen capacity occurred at approximately CT 8 (clade A = 10.24 ± 0.5, D = 20.37 ± 0.65, C = 11.9 ± 1 mg L^−1^). Under LL, the amplitude decreased and the free-run time period became significantly shorter: 23.5 ± 0.15 h for clades A and C, and 24.3 ± 0.2 h for clade D (one-way ANOVA followed by Tukey HSD, *p* < 0.01), with minimum and maximum time points similar to those for the LD cycles (clade A = 9.47 ± 0.24, D = 15.66 ± 0.24, C = 10.42 ± 0.17; figure 2*b*; electronic supplementary material, S1, S2). The oscillations in oxygen evolution corresponded to variations in the maximum quantum yield of PSII measured for both free-living and *in-hospite* *Symbiodinium.* Clade A cultures sampled at 4 h intervals over 2 days of LD displayed a repeating pattern with maximal quantum yields occurring before first light at ZT 0 and minimum quantum yields recorded at midday (ZT 8; *F*_v_/*F*_m_ values ranging between 0.24 and 0.35; figure 3*a*). The daily oscillations continued for two consecutive days of LL, showing similar fluorescence values at similar time points (figure 3*a*; one-way ANOVA followed by Tukey HSD, *p* < 0.01). In addition, the calculated *σ*PSII values for cultured *Symbiodinum* were approximately aligned to the pattern of oxygen evolution concentration, peaking at ZT 8 and CT 8,12 during both LD and LL (figure 3*b*; one-way ANOVA followed by Tukey HSD, *p* < 0.05). Within the natural environment of the host–symbiont association, changes in quantum yields (measured using the PAM method) showed similar temporal trends with efficiencies for ETR, with high values occurring in the first half of the day between CT 0 and 6 under LL conditions. The *F*_v_/*F*_m_ values revealed the same time point as expected in the cultures for the minimum value (CT 10) and the recovery (CT 14; electronic supplementary material, figure S3*a*,*b*). To further elucidate more rhythmic photosynthetic apparatus components governed by the free-living algae timekeeper, an array of pigment concentrations were measured using HPLC during 2 days of LD cycles, followed by 2 days of LL, with sampling intervals of 4 h. Quantification of chlorophyll *a*, accessory pigments (chlorophyll *c* and peridinin) and protective (diatoxanthin and diadinoxanthin) pigments revealed diel fluctuations with maximum concentrations occurring between ZT 8 and ZT 12 during the LD cycle. Under LL conditions, the maximum values phase-shifted on the first day of LL in most of the pigments towards CT 12 (figure 4*a*–*e*). The xanthophyll cycle carotenoids ratio (diatoxanthin/(diatoxanthin + diadinoxanthin)) was rhythmic during the first 48 h under the LD cycle, showing clear peaks at approximately ZT 4 each day, but arrhythmic under LL conditions (figure 4*f*).

**Figure 5.**
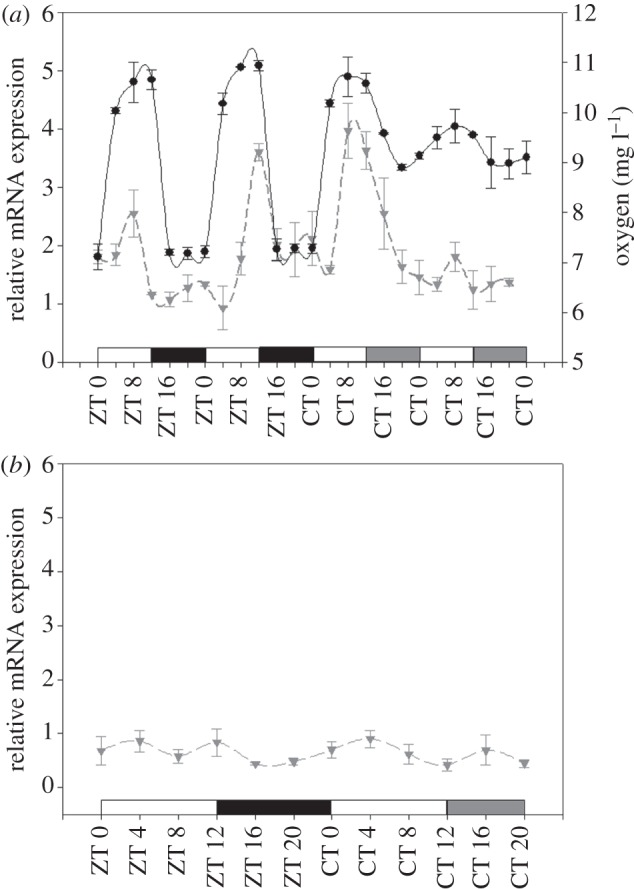
qPCR assays. (*a*) Temporal relative mRNA expression of the oxygen-evolving enhancer (*OEE1*, inverted triangles) gene (*n* = 3 ± s.d., per time point) and oxygen evolution (black circles; *n* = *4* ± s.d., per time point) measured using *Symbiodinium* clade A under 2 days of LD cycles followed by 2 days of LL. (*b*) Relative mRNA expression of the *PsbA* gene during LD cycle followed by LL cycle. The white bars indicate light periods, the black bars indicate dark periods and the grey bars indicate subjective darkness.

Finally, we measured the temporal expression of the *OEE1* gene using real-time PCR on clade A cultures. As *OEE1* is part of the PSII complex responsible for the oxidation of water molecules, we decided to further examine the association between the dissolved oxygen measurements and the pattern of *OEE1* gene expression. *OEE1* mRNA expression levels were higher during the light periods: expression increased between ZT 0 and ZT 8, and decreased thereafter, with minimum expression levels during the subjective night (one-way ANOVA followed by Tukey HSD, *p* < 0.01). The same expression pattern was observed during the LL period (one-way ANOVA followed by Tukey HSD, *p* < 0.05). The temporal pattern of *OEE1* expression reflected the measurements of oxygen evolution in the *Symbiodinium* clade A culture under the same experimental conditions (figure 5*a*). As a reference gene, we used the *PsbA* gene that is known to possess constant mRNA expression levels during LD and LL in dinoflagellates [38].

## 4. Discussion

Photosynthesis, found to be controlled by an endogenous clock in many photosynthetic organisms [12,39], is the most important process in autotrophs, from cyanobacteria to higher plants. In this study, all of the photosynthetic parameters investigated demonstrated diel rhythmicity during LD periods. Our results also reveal a clear periodicity in various photosynthetic parameters under constant artificial light with no alteration in the light spectrum or light intensities. Thus, the rhythmicity was maintaining daily oscillations with a free-run cycle when conditions switched from LD cycles to LL conditions. The rhythms of photosynthesis in *Symbiodinium* show circadian pattern and fulfil the three characteristics thereof: first, the rhythm is kept under constant conditions of light; second, light can entrain the rhythm, whereas different light spectrums change the entrainment of the cycle [40]; and third, our former work shows the ability of *Symbiodinium* in culture as well as inside the coral tissue to compensate for temperature changes and maintain the rhythm under LD and LL [41]. The LL free-run period presented here differs from the LD period mainly in the magnitude of the oxygen evolution. These findings identify the presence of an endogenous oscillator in *Symbiodinium* algae that regulates photosynthesis as an output pathway downstream to the clock pacemaker. As opposed to the well-studied dinoflagellate *Lingulodinium*, little is known about the diel/circadian rhythm of *Symbiodinium* in cell culture or as symbiotic counterparts in coral tissues or other marine invertebrates. The few reports concerning circadian rhythm that imply the presence of an endogenous clock in *Symbiodinium* were related to a motility pattern in algae freshly isolated from corals [10,42,43], to carbon fixation [44] and to *Symbiodinium* cell division, which peaked during the dark periods and showed no rhythmicity during stationary growth stage [43]. Therefore, during our experiments, cell concentrations were kept constant and measurements were performed on cultures during their stationary stage to avoid oscillations related to cell division and growth. The ubiquity of oxygen production in *Symbiodinium* was also observed when measurements were performed on three different cell-cultured clades (A, C, D), thus demonstrating a diel photosynthetic rhythm (figure 2*b*). In addition, all three clades presented a shorter free-run cycle of oxygen production under LL conditions. The ability of different *Symbiodinium* clades to conserve circadian rhythmicity reveals that, like other algal species [45,46], the rhythm of oxygen evolution remains close to a 24 h cycle. Thus, the photosynthetic diel rhythm is not genotype-dependent.

In symbiotic reef-building corals, the association enables the high rates of calcification that are characteristic of reef-building corals; however, the presence of the symbionts is a double-edged sword. Dramatic diurnal physiological changes occur as a consequence of the coral's obligate association with photosynthetic endosymbionts (*Symbiodinium*)*.* Photosynthesis by *Symbiodinium* results in the exposure of coral tissue to over-saturating levels of oxygen in the day, but nearly hypoxic levels at night when respiration dominates [47]. This physiological complexity enables the coral/host to control the cell division and growth of its autotrophic associates in its tissue, most likely as result of evolution [48,49]. Therefore, our findings show that photosynthesis is circadian in both the symbiotically associated *Symbiodinium* form and *Symbiodinium* cultures (figure 1*a*,*b*). These findings suggest that unlike other regulation such as cell proliferation and motility, the symbiont circadian system is probably independent and unaffected by the host's circadian rhythms. As for other photosynthetic parameters, our findings contrast with earlier work that showed constant photosynthetic rates throughout the day in *S. pistillata* corals exposed to continuous and constant irradiance [50], probably because the entrainment was not sufficiently satisfactory to maintain the diel rhythm in those experiments.

The fluorescence measurements of the maximum quantum yield for both *Stylophora* corals (see the electronic supplementary material, figure S3) and *Symbiodinium* cultures (figure 3*a*) using constant artificial light showed diel oscillations, with *F*_v_/*F*_m_ values decreasing at noon (ZT 8 under LD and CT 8 under LL; figure 3*a*). These results are consistent with previous results of quantum yields of corals during an ambient light cycle [51,52]. The results also match similar findings reported for higher plants [53]. During the LL cycle, the circadian rhythm was the same as found in the LD cycle, but with a lower quantum yield value at CT 0 compared with ZT 0. Because the *F*_v_/*F*_m_ value of a dark-adapted sample was proportional to the fraction of reaction centres capable of converting absorbed light to photochemical energy [54], the *F*_v_/*F*_m_ values can be correlated to photodamage and the photoprotective process as part of the photosynthesis apparatus. In this case, the decline of the *F*_v_/*F*_m_ values between ZT 0 and ZT 4 under LD conditions is characterized by a decrease in *F*_0_, which indicates a photoprotective process. Nonetheless, the continuous decline of the *F*_0_ value during the free-run period under LL conditions, and the absence of a dark-repairing refractory period implies an accumulation of photodamage in PSII and the degradation of the D1 protein [55]. As part of our effort to reveal the components of the photosynthetic apparatus controlled by the endogenous circadian clock, we found that the pigments' content are rhythmic (figure 4). Chlorophyll *a*, chlorophyll *c* and peridinin pigments showed daily oscillation maxima at approximately ZT 8, whereas diatoxanthin and diadinoxanthin revealed maximum at ZT 4 during the LD cycle. This pattern of diel oscillation continued when cultures were transferred to constant LL conditions, but presented a phase shift for the time of maximum cell pigment content, for photosynthetic pigments to CT 12 for the first day under LL and recovery to CT 8 for the next day, and for the photoprotective pigments from ZT 4 to CT 8 for the LL period. Previous works have not reported the circadian rhythmicity of pigment content [13,56] or shown diel oscillation in cell pigments that might have resulted from cell division controlled by an endogenous clock [57]. The xanthophyll cycle is an important protective mechanism against photodamage, and its ratio (diatoxanthin/(diatoxanthin + diadinoxanthin)) influences the fluorescence yield, *F*_v_/*F*_m_ [51]. Here, the xanthophyll ratio oscillated under LD conditions and reached maximum values between midday and early afternoon, and then decreased until morning. This temporal fluctuation in the xanthophyll ratio correlates with light exposure time as a mechanism of dissipation of excitation energy, as reported by Brown *et al.* [51], who showed inverse correlations between the xanthophyll ratio and *F*_v_/*F*_m_ values in *Goniastrea aspera* coral. The cycle of the xanthophyll ratio was not observed during the LL period despite the diel oscillation pattern observed in the pigments' content. During the LL period, the xanthophyll ratio increased from the beginning of the first day and remained almost constant during the LL cycle. The absence of circadian rhythmicity observed in xanthophyll during the LL period is most likely owing to the accumulation of reactive oxygen species, or other photo-induced damages that occurred in the photosynthetic apparatus that masked the actual clock pacemaker and obscured the rhythm occurring under LD. This finding indicates that other factors aside from the photoprotective pigments are most probably responsible for the endogenous rhythm of *F*_v_/*F*_m_.

Many physiological studies of algae have attempted to define photosynthesis as a circadian phenomenon, as discussed in the review by Prezelin & Sweeney [45]. The observation of periodicity in the chloroplast shape, thylakoid orientation, periodicity in photosynthetic irradiance curve, photosystem activity, and pigment levels and respiration [58–60] led some researchers to suggest that photosynthesis is regulated owing to the interaction with the photosystem embedded in the thylakoid membrane [45]. This conclusion is in contrast to research that suggested that the rhythmicity is regulated by the dark reactions of photosynthesis [61]. Others have suggested that changes in electron flow through PSII cause photosynthetic circadian rhythmicity in *Lingulodinium* [46] and *Scenedesmus* [62].

Photosynthetic circadian rhythmicity can be alternatively explained by gene regulation, which is driven by the core molecular clock pacemaker. An array of genes related to the photosynthetic process in *Karenia brevis* (dinoflagellate) [63] and in *Arabidopsis* [4,5] were shown to be under the control of the circadian oscillator. These genes include those for the PSI and PSII reaction centre, Psad1, light harvesting complex A and light harvesting complex B, and chlorophyll-binding proteins [4,64–65]. Our molecular work reveals daily oscillations in the *OEE1* gene (most probably downstream to the *Symbiodinium* core oscillator), which is part of the oxygen-evolving enhancer complex (figure 5*a*). This complex is part of the PSII complex, which was found to control the oxidization of water molecules. The removal of the *OEE1* results in both the loss of oxygen evolution and the reduction of manganese ions bound to the thylakoid membrane [66]. In *Chlamydomonas reinhardtii*, the *OEE1* protein is essential for oxygen evolution and the stability of PSII [67]. Gene oscillation during the LD cycle, which continues under LL conditions, was found to correlate with oxygen evolution in *Symbiodinium* under the same conditions. *OEE1* was also reported to be rhythmic in tomato seedlings, with maximum expression between 12:00 and 18:00 and with minor changes in mRNA expression compared with CAB mRNA expression [65]. In contrast to the rhythmic *OEE1* expression, the *PsbA* gene revealed a constant diel pattern both under LD as well as under LL conditions, and showed the same diel pattern that was observed for mRNA expression levels in former research [38], suggesting that *PsbA* is not involved directly in mediating the oxygen evolution rhythm [38]. Our results indicate that the circadian clock mediates the photosynthesis of *Symbiodinium* both in unicellular cultured algae and in symbiosis with corals. Considering that corals have maintained a stable partnership with their symbionts since the Permian mass extinction, subtle physiological and molecular adaptations are to be expected from both partners. Whereas, some of the results described here may reflect unique requirements of the coral/algae association (e.g. high oxygen levels in the coral tissue during the day), others may be more general and resemble patterns in higher lineages [68]. Over evolutionary time, the expression of functionally related processes, such as photosynthesis, can become coupled with global regulatory systems, such as the circadian machinery. The *Stylophora*/dinoflagellate association not only illustrates the evolutionary flexibility of circadian regulatory systems, but also provides a paradigm for further understanding the synchronization of two circadian clocks systems in animal/algal symbioses. One hypothesis to explain how circadian clocks arose is embedded in the symbiotic fusion of a primitive bacterium with a more complex bacterium host cell to form the first eukaryote, a process that required metabolic coordination and synchronization in both partners. Thus, an understanding of the synchronization of circadian clocks in symbiotic basal metazoans, such as corals, will be of particular interest in revealing the evolutionary origin of circadian clocks.
